# Combining stereotactic body radiotherapy with camrelizumab for unresectable hepatocellular carcinoma: a single-arm trial

**DOI:** 10.1007/s12072-022-10396-7

**Published:** 2022-08-24

**Authors:** Jian-Xu Li, Ting-Shi Su, Wen-Feng Gong, Jian-Hong Zhong, Liu-Ying Yan, Jie Zhang, Li-Qing Li, Mei-Ling He, Rui-Jun Zhang, You-Qin Du, Xiao-Ting Wang, Shi-Xiong Liang, Bang-De Xiang

**Affiliations:** 1 Department of Radiation Oncology, Guangxi Medical University Cancer Hospital, Nanning, 530021 China; 2 Department of Hepatobiliary Surgery, Guangxi Medical University Cancer Hospital, Nanning, 530021 China; 3 Department of General Affairs, Guangxi Medical University Cancer Hospital, Nanning, China

**Keywords:** Anti-PD1 monoclonal antibody, Camrelizumab, Clinical trial, Efficacy, Hepatocellular carcinoma, Objective response rate, Palliative, Safety, Stereotactic body radiotherapy, Unresectable

## Abstract

**Purpose:**

Stereotactic body radiotherapy (SBRT) may have significant immunomodulatory effects that enhance tumor response to immune checkpoint inhibitors. This phase 2 clinical trial was conducted to evaluate the safety and efficacy of combining palliative SBRT with camrelizumab (an anti-PD1 monoclonal antibody) in patients with unresectable hepatocellular carcinoma (uHCC).

**Methods:**

Patients with uHCC, Child–Pugh A/B liver function, and at least one measurable lesion were enrolled between April 2020 and August 2022. Patients were administered 200 mg camrelizumab intravenously from the first day of palliative SBRT and then every 3 weeks. Palliative SBRT was delivered daily over five fractions per week, with a dose range of 30–50 Gy. The primary endpoints were objective response rate (ORR) and safety. This trial was registered at ClinicalTrials.gov (NCT04193696).

**Results:**

Twenty-one patients were enrolled; the median radiation dose was 40 Gy, and the median number of cycles of camrelizumab was five. The ORR was 52.4%. After a median follow-up of 19.7 months, the median progression-free and overall survival were 5.8 and 14.2 months, respectively. The overall survival probability was 85.7% at 6 months, 76.2% at 9 months, and 59.9% at 12 months. All grade 3 treatment-related adverse events (TRAEs) occurred in five patients (23.8%) and were manageable. No grade 4/5 TRAEs were observed.

**Conclusion:**

Palliative SBRT plus camrelizumab showed promising antitumor activity against uHCC. Toxicities were manageable with no unexpected safety issues. This study provides evidence of a new therapeutic method for the treatment of uHCC.

**Supplementary Information:**

The online version contains supplementary material available at 10.1007/s12072-022-10396-7.

## Introduction

Hepatocellular carcinoma (HCC) is the third most common cause of cancer-related deaths worldwide and ranked seventh in incidence in 2020 [1]. Most patients present with unresectable disease with a poor prognosis [2, 3]. Lenvatinib and sorafenib are recommended as the first-line treatments for these patients, with an objective response rate (ORR) of only 2.0–18.8% [4–6]. The second-line treatment, regorafenib, extended the ORR to 11% for patients who should progress with sorafenib treatment [7]. However, the treatment outcomes in patients with unresectable hepatocellular carcinoma (uHCC) remain unclear. There are many serious adverse reactions, including diarrhea, weight loss, hand-foot syndrome, and hypophosphatemia [4–7].

The development of immune checkpoint inhibitors (ICIs) has opened a new era in the treatment of uHCC. The ORR was 14–17% in pretreated uHCC patients treated with camrelizumab, nivolumab, and pembrolizumab, which are monoclonal antibodies against programmed cell death ligand 1 (anti-PD1) [8–10]. With the rapid improvement in radiotherapy technology and equipment, the efficacy of radiotherapy for uHCC has significantly improved in recent years. The application of hypofractionated three-dimensional conformal radiotherapy (RT) in the treatment of HCC without extrahepatic metastasis can achieve an overall survival rate of 65% and 33% at 1 and 3 years, respectively [11]. Stereotactic body radiotherapy (SBRT) leads to better local control with a shorter treatment duration and fewer costs [12]. The National Comprehensive Cancer Network guidelines recommend RT as the standard treatment for uHCC [13].

SBRT can promote the host antitumor immune response and enhance the efficacy of cancer immunotherapy [14]. RT combined with anti-PD1 enhanced the abscopal effect in C57BL/6 mice with HCC [15]. In recent years, RT combined with immunotherapy, as a new therapeutic method, has achieved certain efficacy in many cancers, such as non-small cell lung cancer [16], malignant pigmented tumor [17], and prostate cancer [18]. Theoretically, RT combined with anti-PD1 may also produce a synergistic antitumor effect on uHCC. We conducted this phase II clinical trial to evaluate the safety and efficacy of combining palliative SBRT with camrelizumab in patients with uHCC.

## Materials and methods

### Study design and patients

The trial was an open-label, single-arm clinical study registered at ClinicalTrials.gov (NCT04193696). Written informed consent was obtained from all patients. The study was performed according to the ethical guidelines of the Declaration of Helsinki and was approved by the Ethics Committee of Guangxi Medical University Cancer Hospital (KS2019(209)).

Patients were recruited on-site by clinic staff. Data were collected from the electronic charting system of the hospital and patient follow-ups. Eligible patients were 18–70 years of age and had uHCC. All diagnoses were confirmed histologically, cytologically, or clinically based on the American Association for the Study of Liver Diseases criteria [19]. Patients had an Eastern Cooperative Oncology Group performance status (ECOG PS) of 0 or 1 and at least one measurable lesion according to the modified Response Evaluation Criteria in Solid Tumors (mRECIST) [20] and version 1.1 of the Response Evaluation Criteria in Solid Tumors (RECIST v1.1) [21], with Barcelona Clinic Liver Cancer (BCLC) stage B or C classification and Child–Pugh class A or B scale. The patients had previously failed or relapsed after receiving therapy other than immunotherapy checkpoint inhibitors, had stopped using them for more than 20 days prior to baseline screening, and had previously received regional treatment for HCC (including radiofrequency ablation, percutaneous ethanol or acetic acid injection, cryotherapy, high-intensity focused ultrasound, hepatic arterial chemoembolization, and hepatic arterial embolization) with definite progression in the local treatment area according to RECIST v1.1. All patients had adequate liver function (bilirubin ≤ 1.5 times the upper limit of normal (ULN), and aspartate aminotransferase (AST) and alanine aminotransferase (ALT) ≤ 3 times ULN), hemoglobin ≥ 90 g/L, platelet count ≥ 60 × 10^9^/L, and absolute neutrophil count ≥ 1.5 × 10^9^/L. The key exclusion criteria were history of immunotherapy, RT within 6 months before the first administration, active autoimmune diseases requiring systemic treatment, and active infections.

### Treatment

All patients underwent computed tomography (CT) scans for SBRT planning, and the images were acquired at a 2.5–5 mm slice thickness in free quiet breathing mode. Vacuum body cushions and thermoplastic body masks were used for patient immobilization. All the target volumes and organs at risk (OARs) were contoured in the MIM 6.8 system; gross target volume (GTV) was determined by imaging examinations. CT-magnetic resonance imaging (MRI) fusion for lesions in the liver and CT-positron emission tomography (PET-CT) fusion for extrahepatic lesions were performed to clearly show the lesion. For patients with multiple lesions, as many lesions as possible were chosen for SBRT if tolerated at the discretion of the radiation oncologists. The GTV was expanded to 5–10 mm to establish the planning GTV (PGTV). The palliative radiation dose to PGTV ranged from 30–50 Gy in 10 fractions. The SBRT plans were designed using the Monaco treatment planning system version 5.1 and performed using volumetric-modulated arc therapy. The OARs were the priority constraints on PGTV for treatment planning. Palliative SBRT was delivered daily over five fractions per week using a 6 MV X-ray linear accelerator (ELEKTA Versa-HD). Cone-beam CT images were used to correct the positions.

The camrelizumab regimen, including dose, method of injection, and duration of treatment, was developed according to the RESCUE trial [22] and the guideline provided by the manufacturer. Briefly patients were administered 200 mg camrelizumab intravenously over 30 min from the first day of SBRT and then every 3 weeks until disease progression or intolerable toxicity. Camrelizumab could be delayed for up to 12 weeks throughout the study, but the dose could not be adjusted. Patients whose disease had progressed were permitted to continue camrelizumab if the researchers judged that they would benefit from and tolerate treatment.

### Evaluation of efficacy and safety

RECIST v1.1 and mRECIST were used to evaluate tumor responses in target and nontarget lesions by two investigators. The first imaging evaluation was performed during the third camrelizumab session at about 6 weeks after the start of SBRT, the second one at about 12 weeks after SBRT, and then every 3 months. The primary endpoint was safety, and the ORR was defined as the sum rate of complete remission (CR) and partial remission (PR). The secondary endpoints were overall survival (OS) measured from the day of informed consent to the day of death; progression-free survival (PFS), defined as the time from informed consent until disease progression or death; and disease control, defined as the sum of CR + PR + stable disease (SD). All participants were followed up monthly for progression and survival status. All adverse events (AEs) and treatment-related adverse events (TRAEs) were recorded from the first SBRT until 90 days after the last camrelizumab injection, according to the Common Terminology Criteria for Adverse Events of the National Cancer Institute v5.0.

### Statistical analysis

Assuming an ORR in 50% of patients treated with combination of SBRT and camrelizumab, 21 patients could provide 80% power to ensure the lower boundary and 0.05 alpha to reject a true null hypothesis in a one-sample exact binomial test at the significance level of 0.05 vs. 18.8% treated with lenvatinib. We conducted sample size statistical analyses using the PASS v15 software (NCSS, Kaysville, UT, USA).

Baseline demographic and clinical characteristics are presented as mean ± standard deviation, median (range), or *N* (%). The ORR and disease control rate (DCR) were calculated with 95% confidence intervals (CI) using the Clopper–Pearson method. PFS, time to response rate (TTR), and OS were estimated using the Kaplan–Meier method. TTR was analyzed in patients with a confirmed CR or PR. The duration of follow-up was calculated using the reverse Kaplan–Meier estimate of OS. All statistical analyses were performed using IBM SPSS software (ver. 26.0 SPSS Inc., Chicago, IL, USA).

## Results

### Patients and treatment

**Fig. 1:**
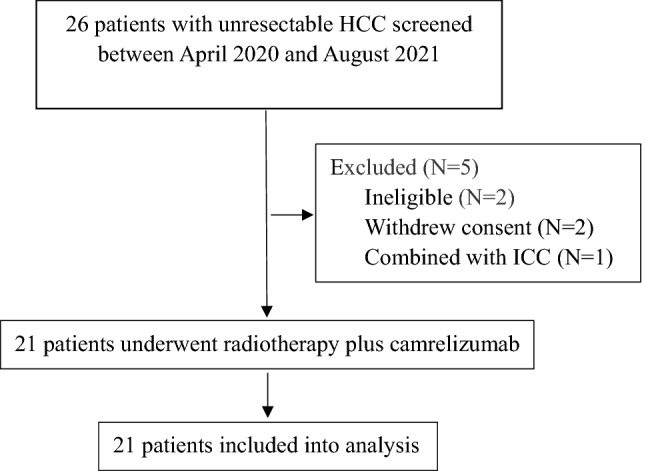
Patient selection flow. *HCC* hepatocellular carcinoma, *ICC* intrahepatic cholangiocarcinoma

From April 3, 2020 to January 21, 2021, 26 patients were screened for eligibility (Fig. 1), and 21 patients were enrolled in the study (the analysis set). At the cutoff date (May 1, 2022), the median follow-up of all patients in the analysis set was 19.7 months (95% CI 17.4–22.0), and patients received a median of 5 (range 2–27) cycles of camrelizumab. All 21 patients with more than 78 lesions (one patient with too many uncountable pulmonary metastases was counted as 4, and four patients with more than 3 lesions were counted as 4) received palliative SBRT for 45 lesions including 24 intrahepatic lesions and 21 extrahepatic lesions. Due to the high costs of the treatment, 3 of the 4 patients with SD as best response did not continue camrelizumab, along with 7 of the 10 patients with PR as best response by RECIST v1.1. After disease progression, of all the patients, 4 with ECOG PS 0–1 and without taboos refused any subsequent treatment, 1 underwent palliative surgical resection, 1 received sorafinib, 3 received apatinib, and 5 received lenvatinib.

As shown in Table 1, the ages of the participants ranged from 31 to 69 years, with a median age of 54 years, and a male probability of 90.5%. Twenty patients were diagnosed with BCLC-C and one with BCLC-B, with 85.7% having chronic hepatitis B virus (HBV) etiology and 52.4% presenting with radiographic liver cirrhosis. Up to 19 patients (90.5%) had multiple lesions, including 10 (47.6%) with more than or equal to 4 lesions, and 10 (47.6%) had received two or more lines of previous treatments. Among those 3 patients (14.3%) who had undergone previous systemic therapy, 2 received sorafenib and 1 received apatinib before the trial.

**Table 1:** Baseline demographic and clinical characteristics

Characteristic	Value
Sex, male	19 (90.5)
Age, year	54 (31–69)
Hepatitis B virus infection, present	18 (85.7)
Liver cirrhosis, present	11 (52.4)
ECOG PS	
0	14 (66.7)
1	7 (34.3)
Total bilirubin, μmol/L	15.3 ± 4.4
Albumin (g/L)	36.9 (24.8–41.6)
Child–Pugh grade	
A	20 (95.2)
B	1 (4.8)
ALBI grade	
1	5 (23.8)
2	15 (71.4)
3	1 (4.8)
Alpha fetoprotein, ≥ 400 ng/ml	10 (47.6)
Maximum tumor size, (cm)	7.0 ± 4.0
Tumor number	
1	2 (9.5)
2	6 (28.6)
3	3 (14.3)
≥ 4	10 (47.6)
Macrovascular invasion, present	10 (47.6)
Extrahepatic metastasis, present	14 (66.7)
BCLC stage	
B	1 (4.8)
C	20 (95.2)
Dose (Gy)	40 (30–50)
Gross tumor volume, (ml)	205 (11–1471)
Prior therapy	
TACE	9 (42.9)
Hepatectomy	16 (76.2)
RFA	6 (28.6)
Systemic therapy	3 (14.3)

Data are mean ± standard deviation, median (range) or *N* (%)

*ALBI* the Albumin-Bilirubin grade, *BCLC* Barcelona Clinic Liver Cancer, *ECOG PS* Eastern Cooperative Oncology Group performance status, *RFA* radiofrequency ablation, *TACE* trans-arterial chemoembolization

### Efficacy

**Fig. 2:**
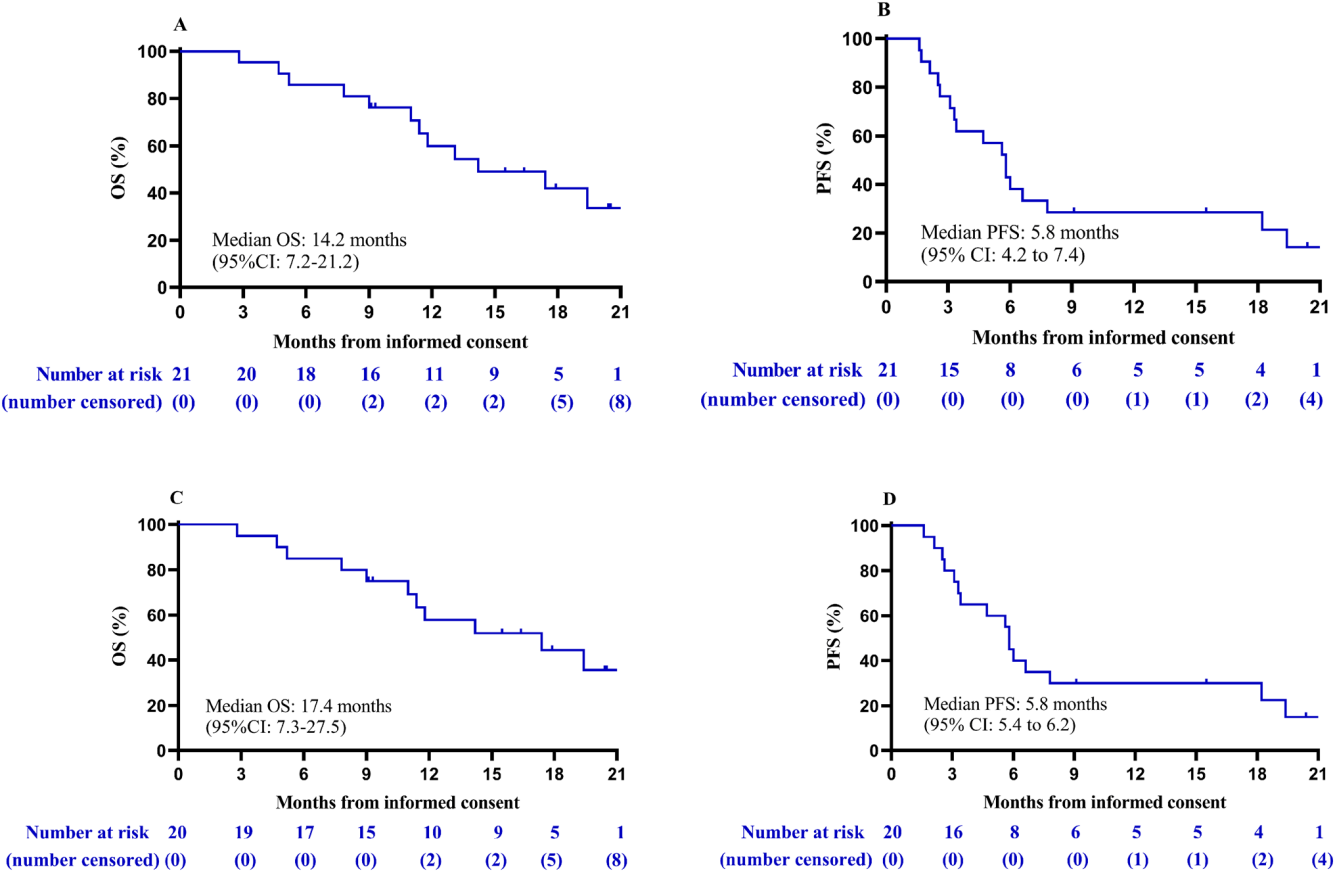
Kaplan–Meier analysis of overall and progression-free survival. **a** Overall and **b** progression-free survival for all patients, **c** overall and **d** progression-free survival for patients with Barcelona Clinic Liver Cancer stage C disease

A summary of efficacy outcomes after a median follow-up of 19.7 months is shown in Table 2. The ORR was 52.4% according to RECIST v1.1 and mRECIST. CR was observed in one patient (4.8%) according to RECIST v1.1 and two patients (9.5%) according to mRECIST. PR was observed in 10 patients (47.6%) according to RECIST v1.1 and 9 patients (42.9%) according to mRECIST. Fourteen patients (66.7%) achieved disease control according to the RECIST 1.1 and mRECIST. The median OS was 14.2 months (95% CI 7.2–21.2, Fig. 2a, Table 2). The OS probability was 85.7% (95% CI 62.0–95.2) at 6 months, 76.2% (95% CI 51.9–89.3) at 9 months, and 59.9% (95% CI 35.3–77.7) at 12 months. The median PFS was 5.8 months (95% CI 4.2–7.4, Fig. 2b, Table 2). For the other secondary efficacy endpoints, the median time to response was 2.1 months (95% CI 1.9–2.3). For the 20 patients with BCLC stage C disease, after a median follow-up of 19.4 months, the ORR was 55.5%, the median OS was 17.4 months (95% CI 7.3–27.5, Fig. 2c, Supplemental Table 1) and the median PFS was 5.8 months (95% CI 5.4–6.2, Fig. 2d, Supplemental Table 1).

**Table 2:** Summary of efficacy outcomes (*N* = 21)

Outcomes	RECIST1.1	mRECIST
Objective response	11 (52.4)	11 (52.4)
Disease control	14 (66.7)	14 (66.7)
Best overall response		
CR	1 (4.8)	2 (9.5)
PR	10 (47.6)	9 (42.9)
SD	3 (14.3)	3 (14.3)
PD	7 (33.3)	7 (33.3)
Progression-free survival, median months (95% CI)	5.8 (4.2–7.4)	
Overall survival, %		
6 months, (95% CI)	85.7 (62.0–95.2)	
9 months, (95% CI)	76.2 (51.9–89.3)	
12 months, (95% CI)	59.9 (35.3–77.7)	
Median overall survival time, months (95% CI)	14.2 (7.2–21.2)	
Time to response, median months (95% CI)	2.1 (1.9–2.3)	

Data are *N* (%, or 95% CI), unless indicated

*CR* complete response, *PD* progressive disease, *PR* partial response, *SD* stable disease

**Fig. 3:**
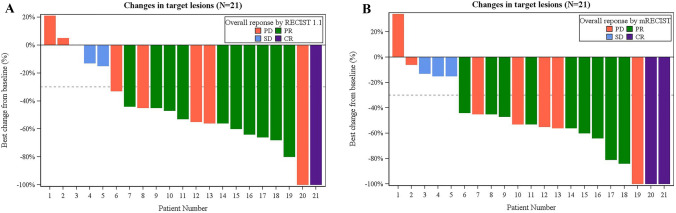
Best percentage change from baseline in sums of diameters of target lesions by RECIST 1.1 (**a**) and mRECIST (**b**). *CR* complete response, *PR* partial response, *SD* stable disease, *PD* progressive disease

The rate of shrinkage of the tumor target lesion is shown by a waterfall plot. Seventeen patients (81.0%) achieved ≥ 30% tumor target lesion shrinkage according to RECIST v1.1 (Fig. 3a) and mRECIST (Fig. 3b) from baseline at one or more radiographic evaluation time points during the entire treatment period. Eighteen (85.7%) and 20 (95.2%) patients achieved tumor target lesion shrinkage from baseline according to RECIST v1.1 and mRECIST, respectively.

One patient (5.3%) with multiple lymph nodes and peritoneal metastases exhibited hyperprogression of the target lesions, and extensive serious malignant hemorrhagic pleural effusion and new target lesions appeared. He developed a continuous fever and died 2.8 months after enrollment because of tumor progression.

### Safety

All TRAEs of any grade are summarized in Table 3. The most common TRAEs of any grade were reactive cutaneous capillary endothelial proliferation (RCCEP) in 17 patients (81.0%), decreased white blood cell count in 16 (76.2%), decreased hemoglobin in 14 (66.7%), decreased neutrophil count in 13 (61.9%), decreased albumin in 11 (52.4%), increased AST in 11 (52.4%), increased ALT in 10 (47.6%), and asthenia in 10 (47.6%).

**Table 3:** Treatment-related adverse events

Adverse events	Any grade	Grade 1	Grade 2	Grade 3
All events	19 (90.5)	19 (90.5)	16 (76.2)	5 (23.8)
Serious events	1 (4.8)	0 (0)	0 (0)	1 (4.8)
Events leading to discontinuation	4 (19.1)	0 (0)	3 (14.3)	1 (4.8)
RCCEP	17 (81.0)	15 (71.5)	2 (9.5)	0 (0)
Decreased white blood cell	16 (76.2)	7 (33.3)	9 (42.9)	0 (0)
Decreased hemoglobin	14 (66.7)	8 (38.1)	4 (19.1)	2 (9.5)
Decreased neutrophil count	13 (61.9)	9 (42.9)	3 (14.3)	1 (4.8)
Decreased albumin	11 (52.4)	7 (33.3)	4 (19.1)	0 (0)
Increased AST	11 (52.4)	10 (47.6)	1 (4.8)	0 (0)
Asthenia	10 (47.6)	10 (47.6)	0 (0)	0 (0)
Increased ALT	10 (47.6)	8 (38.1)	2 (9.5)	0 (0)
Increased γ-glutamyltransferase	7 (33.3)	6 (28.6)	0 (0)	1 (4.8)
Nausea	5 (23.8)	5 (23.8)	0 (0)	0 (0)
Decreased platelet count	4 (19.1)	0 (0)	3 (14.3)	1 (4.8)
Increased blood bilirubin	4 (19.1)	2 (9.5)	2 (9.5)	0 (0)
Anxiety	4 (19.1)	4 (19.1)	0 (0)	0 (0)
Hypothyroidism	2 (9.5)	2 (9.5))	0 (0)	0 (0)
Fever	2 (9.5)	2 (9.5)	0 (0)	0 (0)
Pneumonia	1 (4.8)	0 (0)	1 (4.8)	0 (0)
Increased ALP	1 (4.8)	0 (0)	1 (4.8)	0 (0)

Data are *N* (%)

*ALP* alkaline phosphatase, *ALT* alanine aminotransferase, *AST* aspartate aminotransferase, *RCCEP* reactive cutaneous capillary endothelial proliferation

Grade 3 treatment-related AEs occurred in five patients (23.8%), including decreased hemoglobin in two patients, decreased neutrophil count in one, increased γ-glutamyltransferase in one, and decreased platelet count in one. Grade 4 or 5 TRAEs were not observed. The adverse events are summarized in Supplemental Table 2. Briefly, more than TRAEs, one patient presented with a grade two food allergy, and one with grade three esophageal and gastric variceal bleeding. Serious AEs were reported in two patients, namely treatment-related decreased platelet count in one and esophageal and gastric variceal bleeding and decreased platelet count in another, who had severe varicose esophagogastric fundus veins at enrollment.

## Discussion

To our knowledge, few prospective clinical trials have reported the safety and efficacy of combining anti-PD1 with SBRT for patients with uHCC. It should be noted that, as shown in Table 1, most patients enrolled in the study had advanced stage disease (95.2% BCLC-C), large tumor burden, high AFP values, and were refractory to multiple prior line therapies with dismal prognosis. The combination treatment was safe and well tolerated, and promising antitumor activity was observed. Combining anti-PD1 therapy with SBRT might be a potential strategy to efficiently treat patients with uHCC.

Qin et al. (2020) reported that Chinese patients with similar baseline demographic and clinical characteristics received camrelizumab treatment. The ORR was 14.7%, and the median PFS was 2.1 months [8]. Camrelizumab has shown remarkable antitumor activity in patients with uHCC. In our study, the ORR (52.7% vs. 14.7%) and PFS (5.8 vs 2.1 months) were much better. Furthermore, in our study, CR was observed in one patient (4.8%) according to RECIST v1.1 and in two patients (9.5%) according to mRECIST. Fourteen patients (66.7%) achieved disease control according to the RECIST v1.1 and mRECIST. Compared with camrelizumab alone, OS probability with the combination treatment was 85.7% vs. 74.4% at 6 months, 76.2% vs. 64.0% at 9 months, and 59.9% vs. 55.9% at 12 months, revealing the therapeutic efficacy of combining anti-PD1 with SBRT. It is worth noting that 10 patients (71.4%) with disease control as the best response received 5 cycles of camrelizumab and did not continue treatment, and 4 with ECOG PS 0–1 and without taboos after disease progression refused any subsequent treatment, which may be an important reason for the decline in the efficacy of combining anti-PD1 with SBRT for uHCC. The efficacy of radiotherapy for uHCC has improved significantly in recent years. Eleni Gkika. reported that the median OS in the treatment of uHCC was 9 months (95% CI 7.7–10.3) [23]. For BCLC-C patients in South Korea, the median OS in the RT group was significantly longer than that in the sorafenib group (7.6 vs 3.8 months, *p* < 0.001) [24]. In this study, the median OS was significantly longer than that observed with SBRT alone. The combination of anti-PD1 and SBRT achieved better antitumor effects than anti-PD1 or SBRT alone in uHCC patients. This is mainly attributed to the phenomenon that non-targeted distant tumors were downsized following RT, known as the “abscopal effect” [25]. RT can gradually cause immunogenic death, such as cell death, which effectively exposes tumor antigens and triggers an immune response throughout the course and several months after RT [26]. RT can reprogram the tumor microenvironment by inducing chemokines and promoting dendritic cell maturation involved in the recruitment of effector T cells. Antigen-educated T cells can home not only into the irradiated but also non-irradiated tumor deposits, and may cause tumor regression [27]. Adding RT to ICI increases the clinical benefit in non-irradiated tumor sites in patients with HCC [28].

Sorafenib is recommended as a first-line treatment for patients with uHCC. The median OS in the sorafenib group was only 6.5 months, and the ORR was only 3.3% in Asia–Pacific uHCC patients with 70.7% HBV infection [6]. In our study, all patients were from China, and 85.7% had a chronic HBV etiology, indicating that they were more prone to develop progressive disease and had poorer prognoses [29]. Other baseline demographic and clinical characteristics were similar. Their clinical outcomes were significantly better than those treated with sorafenib. The REFELECT study showed that lenvatinib was not inferior to sorafenib in untreated patients with uHCC. The ORR in the lenvatinib arm was 18.8% by RECIST v1.1, based on a masked independent imaging review, and that in the sorafenib arm was 6.5% [5]. Better ORR was observed in our study, although most patients were refractory to multiple prior line therapies with dismal prognosis and high rates of chronic HBV infection, meaning that they were more prone to develop progressive disease and had poorer prognoses than those with hepatitis c virus [29, 30]. Combining atezolizumab with bevacizumab improved the efficacy of systemic therapy for uHCC, and the patients had an ORR of 27.3% according to independent assessment with RECIST v1.1 in the IMbrave150 trial [31]. Combining anti-PD1 with SBRT achieved better ORR and similar survival probabilities at 6 months (85.7% vs. 84.8%) and 12 months (59.9% vs. 67.2%). It should be noted that the patients with BCLC-A and B accounted for 17% in the IMbrave150 trial, which is much higher than that in our study (4.8%), and a large proportion of patients without progressive disease failed to remain on camrelizumab. Meanwhile, treatment with atezolizumab plus bevacizumab was too expensive.

As up to 95.2% of patients in this study had BCLC-C, the subgroup data were analyzed. The median OS with BCLC-C was 17.4 months in this study and 5.6 months for the Asia–Pacific patients receiving sorafenib [6]. For the subgroup BCLC-C analysis, the REFELECT study showed the median OS was 11.8 months in the lenvatinib group and 10.3 months in the sorafenib group [5]. This study revealed that the combination of camrelizumab with SBRT showed promising antitumor activity in patients with BCLC-C HCC.

In this study, there were no new or unexpected toxicities resulting from the SBRT plus camrelizumab combination therapy. Grade 3 TRAEs occurred in five patients (23.8%), including two with decreased hemoglobin, one with decreased neutrophil count, one with increased γ-glutamyltransferase, and one with decreased platelet count. The safety of SBRT plus camrelizumab combination therapy in this population was consistent with that of camrelizumab or SBRT alone in previously reported studies [8, 12, 23]. The occurrence rate of grade ≥ 3 TRAEs was less than 57% in patients treated with lenvatinib and 49% in patients treated with sorafenib reported in REFLECT [5], and 56.5% in patients treated with atezolizumab plus bevacizumab reported in IMbrave150 [31]. Serious AEs were reported in two patients (9.5%), including one patient with non-treatment-related esophageal and gastric variceal bleeding. Considering that this patient had severe varicose esophagogastric fundus veins at enrollment, we believe that the serious AEs were not treatment-related side effects.

This study has several limitations. First, the small sample size and nature of this single-arm study were the main limitations. Therefore, the results presented herein should be regarded as preliminary, requiring a larger sample size and phase 3 clinical studies for confirmation. Second, as it was not mandatory for patients to provide tumor samples, the lack of samples for PD-1/PD-L1 detection could be considered a limitation. Third, the patients could not continue camrelizumab because they could not afford it. However, in this case, promising antitumor activity was observed.

In conclusion, the results of this study demonstrated an acceptable safety profile of combining camrelizumab with SBRT for patients with uHCC. Despite the small sample size, we observed preliminary antitumor activity, especially in BCLC-C HCC. Combination treatment has several advantages over other recommended treatments: it incurs less cost, produces less grade ≥ 3 TRAEs, and is easier to accomplish than other treatments while having similar or better antitumor activity. This study provides evidence for a new therapeutic method that combines camrelizumab with SBRT for the treatment of uHCC.

## Supplementary Information

Below is the link to the electronic supplementary material.

Supplementary file1 (DOCX 17 KB)

Supplementary file2 (DOCX 19 KB)

## Data Availability

The data underlying this article will be shared on reasonable request to the corresponding author.
