# Supplementary material for: Combining stereotactic body radiotherapy with camrelizumab for unresectable hepatocellular carcinoma: a single-arm trial
Source: Hepatol Int. 2022 Aug 24;16(5):1179–87. doi: 10.1007/s12072-022-10396-7 (PMC9525355; doi:10.1007/s12072-022-10396-7)
Supplement: Supplementary file 1 — Supplementary file1 (DOCX 17 KB) [file 12072_2022_10396_MOESM1_ESM.docx]

Supplemental Table 1. Summary of Efficacy Outcomes for BCLC-C (N=20).

| **Outcomes** | **RECIST1.1** | **mRECIST** |
| --- | --- | --- |
| Confirmed objective response | 11 (55.0) | 11 (55.0) |
| Disease control | 14 (70.0) | 14 (70.0) |
| Best overall response |  |  |
| CR | 1(5.0) | 2(10.0) |
| PR | 10 (50.0) | 9 (45.0) |
| SD | 3 (15.0) | 3 (15.0) |
| PD | 6 (30.0) | 6 (30.0) |
| Progression-free survival, median months (95% CI) | 5.8 (5.4-6.2) | |
| Overall survival, % |  |  |
| 6-month, (95% CI) | 85.0 (60.4-94.9) | |
| 9-month, (95% CI) | 75.0 (50.0-88.7) | |
| 12-month, (95% CI) | 57.7 (32.7-76.3) | |
| Median months (95% CI) | 17.4 (7.3-27.5) | |
| Time to response, median months (95% CI) | 2.1 (1.9-2.3) | |

Data are N (%, or 95% CI), unless indicated.

CR, Complete response; PD, Progressive disease; PR, Partial response; SD, Stable disease.
