# Supplementary material for: Combining stereotactic body radiotherapy with camrelizumab for unresectable hepatocellular carcinoma: a single-arm trial
Source: Hepatol Int. 2022 Aug 24;16(5):1179–87. doi: 10.1007/s12072-022-10396-7 (PMC9525355; doi:10.1007/s12072-022-10396-7)
Supplement: Supplementary file 2 — Supplementary file2 (DOCX 19 KB) [file 12072_2022_10396_MOESM2_ESM.docx]

Supplemental Table 2. Adverse Events

| **Adverse Events** | **Any Grade** | | **Grade 1** | | **Grade 2** | | **Grade 3** |
| --- | --- | --- | --- | --- | --- | --- | --- |
| All events | 21 (100.0) | 21 (100.0) | | 16 (76.2) | | 6 (28.6) | |
| Serious events | 2 (9.5) | 0 (0) | | 0 (0) | | 2 (9.5) | |
| Events leading to discontinuation | 5 (23.8) | 0 (0) | | 3 (14.3) | | 2 (9.5) | |
| RCCEP | 17 (81.0) | 15 (71.5) | | 2 (9.5) | | 0 (0) | |
| Decreased white blood cell | 17 (81.0) | 8 (38.1) | | 9 (42.9) | | 0 (0) | |
| Decreased hemoglobin | 15 (71.5) | 9 (42.9) | | 4 (19.1) | | 2 (9.5) | |
| Decreased neutrophil count | 13 (61.9) | 9 (42.9) | | 3 (14.3) | | 1 (4.8) | |
| Decreased albumin | 11 (52.4) | 7 (33.3) | | 4 (19.1) | | 0 (0) | |
| Increased AST | 11 (52.4) | 10 (47.6) | | 1 (4.8) | | 0 (0) | |
| Asthenia | 10 (47.6) | 10 (47.6) | | 0 (0) | | 0 (0) | |
| Increased ALT | 10 (47.6) | 8 (38.1) | | 2 (9.5) | | 0 (0) | |
| Increased γ-glutamyltransferase | 7 (33.3) | 6 (28.6) | | 0 (0) | | 1 (4.8) | |
| Nausea | 6 (28.6) | 6 (28.6) | | 0 (0) | | 0 (0) | |
| Anxiety | 5 (23.8) | 5 (23.8) | | 0 (0) | | 0 (0) | |
| Decreased platelet count | 4 (19.1) | 0 (0) | | 3 (14.3) | | 1 (4.8) | |
| Increased blood bilirubin | 4 (19.1) | 2 (9.5) | | 2 (9.5) | | 0 (0) | |
| Hypothyroidism | 2 (9.5) | 2 (9.5)) | | 0 (0) | | 0 (0) | |
| Fever | 2 (9.5) | 2 (9.5) | | 0 (0) | | 0 (0) | |
| Esophagogastric variceal bleeding | 1 (4.8) | 0 (0) | | 0 (0) | | 1 (4.8) | |
| Pneumonia | 1 (4.8) | 0 (0) | | 1 (4.8) | | 0 (0) | |
| Allergy | 1 (4.8) | 0 (0) | | 1 (4.8) | | 0 (0) | |
| Increased ALP | 1 (4.8) | 0 (0) | | 1 (4.8) | | 0 (0) | |

Data are N (%).

ALP, alkaline phosphatase; ALT, alanine aminotransferase; AST, aspartate aminotransferase; RCCEP, reactive cutaneous capillary endothelial proliferation.
